# Squirrel bite—a rare cause of necrotizing soft tissue infection: Case report

**DOI:** 10.1002/ccr3.9099

**Published:** 2024-06-16

**Authors:** Prosper Adjei, Boateng Bosomtwi, Prince Henry Asamoah, Stanley Anenyemele Asasu, Dominic Asante Ohene, Augustina Amoakohene‐Yeboah, Kojo Eduakwa Abban

**Affiliations:** ^1^ Department of Internal Medicine Methodist Hospital Wenchi Ghana; ^2^ Urology Unit Methodist Hospital Wenchi Ghana

**Keywords:** necrotizing fasciitis, necrotizing soft tissue infection, squirrel, squirrel bite

## Abstract

Although squirrel bites are uncommon and generally benign, they have been implicated in the transmission of certain diseases in humans, some of which can be life‐threatening. This report discusses the case of a 27‐year‐old hunter who developed a necrotizing soft tissue infection with vesiculo‐bullous skin lesions after a ground squirrel bite.

## Graphical abstract

Necrotizing soft tissue infection caused by a squirrel bite.

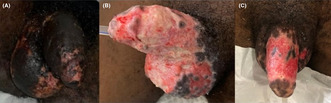

## 1 INTRODUCTION

Domestic animal bites are more common than those involving wildlife. Infections resulting from these bites can lead to severe illness or even death in some instances.^1^ Squirrels are small or medium‐sized rodents. They belong to the family Sciuridae which comprises ground squirrels, tree squirrels and flying squirrels.^2^ Squirrel bites are uncommon and infrequently encountered in medical practice. These rodents have been implicated in the transmission of certain diseases in humans.^3^

Necrotizing soft tissue infection is an infectious process characterized by rapidly progressive soft tissue destruction with associated systemic toxicity.^4^ The epidermis, dermis, subcutaneous tissue, fascia and muscle may be affected.^5^ It is a rare disorder with annual incidence ranging from 0.3 to 15.5 cases per 100,000 population.^6^ Even with optimal treatment, it is associated with a high mortality rate of 25%.^7^ Based on microbial etiology, necrotizing soft tissue infection is mainly classified into polymicrobial (type I) and monomicrobial (type II) infections. Polymicrobial necrotizing soft tissue infection is more common and it is caused by aerobic and anaerobic bacteria. The monomicrobial type is often caused by group A *Streptococcus* and *Staphylococcus aureus*.^4^ Factors associated with an increased risk of necrotizing soft tissue infection include recent trauma, intravenous drug use, diabetes mellitus, steroid use, human immunodeficiency virus (HIV) infection, chronic alcoholism, malnutrition, peripheral vascular disease, obesity, liver cirrhosis, and malignancy.^8^

The clinical manifestations can be varied. Individuals may present with fever, malaise, diarrhea, anorexia and severe pain. Skin necrosis, bullae and ecchymosis can develop in the affected areas of the body. Other clinical features include erythema, edema, and crepitus. Reduced sensation to pain occurs in the affected areas particularly in those with necrotizing fasciitis. This results from thrombosis of small blood vessels and destruction of superficial nerves in the subcutaneous tissue.^4^

Laboratory findings are nonspecific. The Laboratory Risk Indicator for Necrotizing Fasciitis (LRINEC) score is a simple tool consisting of six parameters namely total white cell count, hemoglobin, sodium, glucose, creatinine, and C‐reactive protein. It is used to distinguish necrotizing infections from other soft tissue infections. A score of ≥6 has been reported to have a sensitivity of 92.9% and a specificity of 91.6% for necrotizing soft tissue infection. Its positive and negative predictive values are 92.0% and 96.0%, respectively.^9^, ^10^ The details of the LRINEC scoring system are indicated in Table 1. The mainstay of treatment for necrotizing soft tissue infection involves early surgical debridement of necrotic tissue, initiation of broad‐spectrum empiric antibiotic therapy and hemodynamic support.^11^

**TABLE 1:** Details of LRINEC scoring system.

Laboratory parameter	LRINEC score
Total white blood cell count (per uL)	
<15	0
15–25	1
>25	2
Hemoglobin (g/dL)	
>13.5	0
11–13.5	1
<11	2
Sodium (mmol/L)	
≥135	0
<135	2
Glucose (mg/dL)	
≤180	0
>180	1
Creatinine (mg/dL)	
≤1.6	0
>1.6	2
C‐reactive protein (mg/L)	
<150	0
≥150	4
Total score	<6 (low risk)
	6–7 (intermediate risk)
	≥8 (high risk)

This report discusses a rare case of necrotizing soft tissue infection involving the face, extremities and external genitalia which occurred after a provoked squirrel bite.

## 2 CASE PRESENTATION

### 2.1 Case history and examination

A 27‐year‐old hunter with no significant past medical history was brought to the emergency unit of a district hospital in Ghana after a provoked squirrel bite. Five days prior to the presentation, he went out hunting with his dog which caught a ground squirrel. While attempting to take the squirrel away from the dog, his left little finger was bitten by the rodent. He developed a painful, mild swelling at the affected site. He did not seek immediate medical attention but rather applied topical herbal preparation to the site of the bite. Two days later, he had chills, vomiting, nonbloody diarrhea, and generalized weakness. On the third day after the bite, he woke up with blisters on his face, extensor surfaces of the forearms, palms, knees, dorsum of the feet, scrotum, and penis. The blisters on his genitals later ulcerated. The anterior and posterior parts of his trunk were largely unaffected. This was associated with fever, malaise, and moderate generalized body pain. However, there was no itching, mouth sores, odynophagia, ocular pain, discharge from the eyes, dysuria, epistaxis, or bleeding from any part of the body. He continued to use topical and oral herbal preparations but his symptoms did not improve. He denied a history of recent medication use prior to the onset of his symptoms. He had no drug or food allergies. He neither used intravenous drugs nor steroids. He was a former cigarette smoker but did not drink alcohol.

**FIGURE 1:**
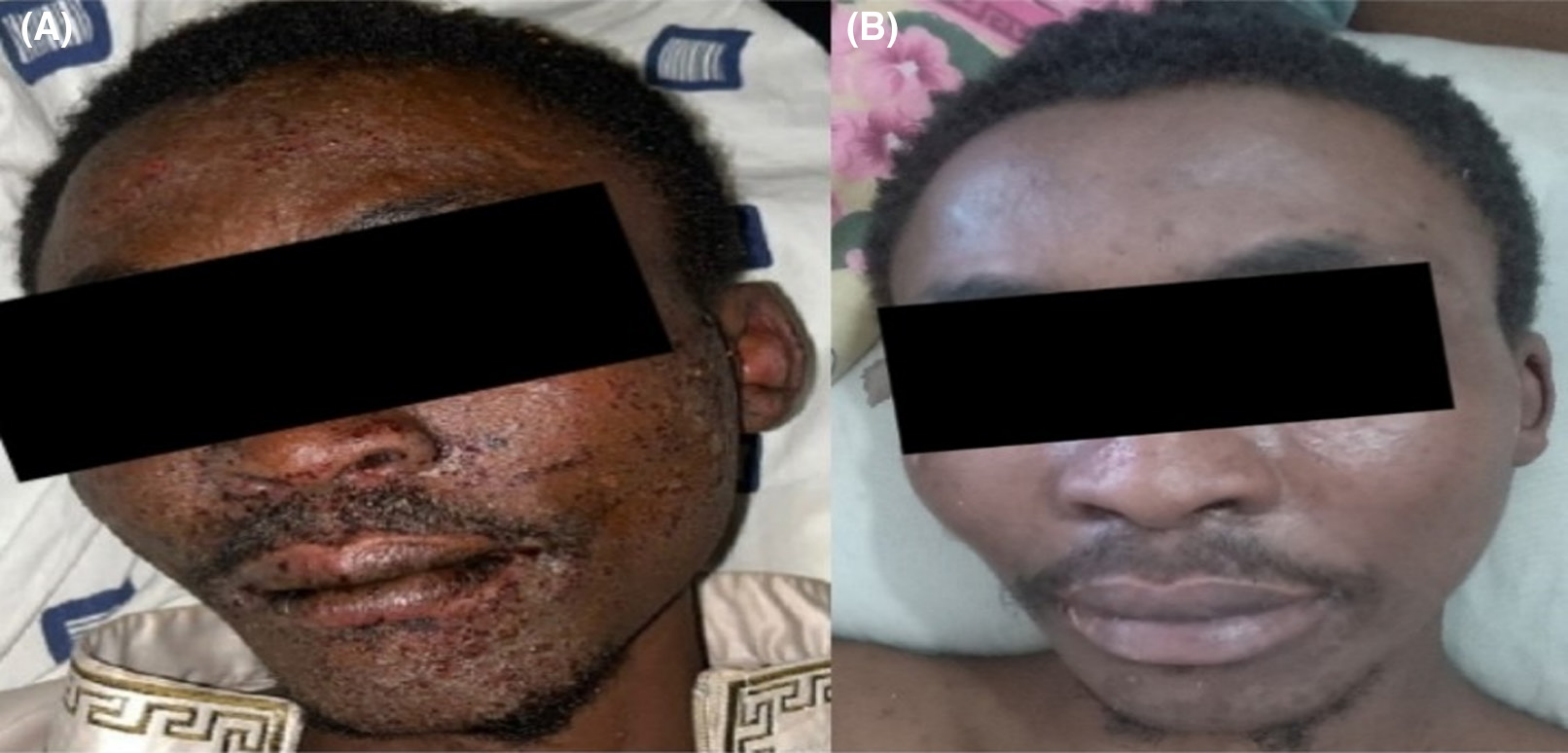
(A) Multiple crusted lesions on the face (B) Complete resolution of facial lesions.

**FIGURE 2:**
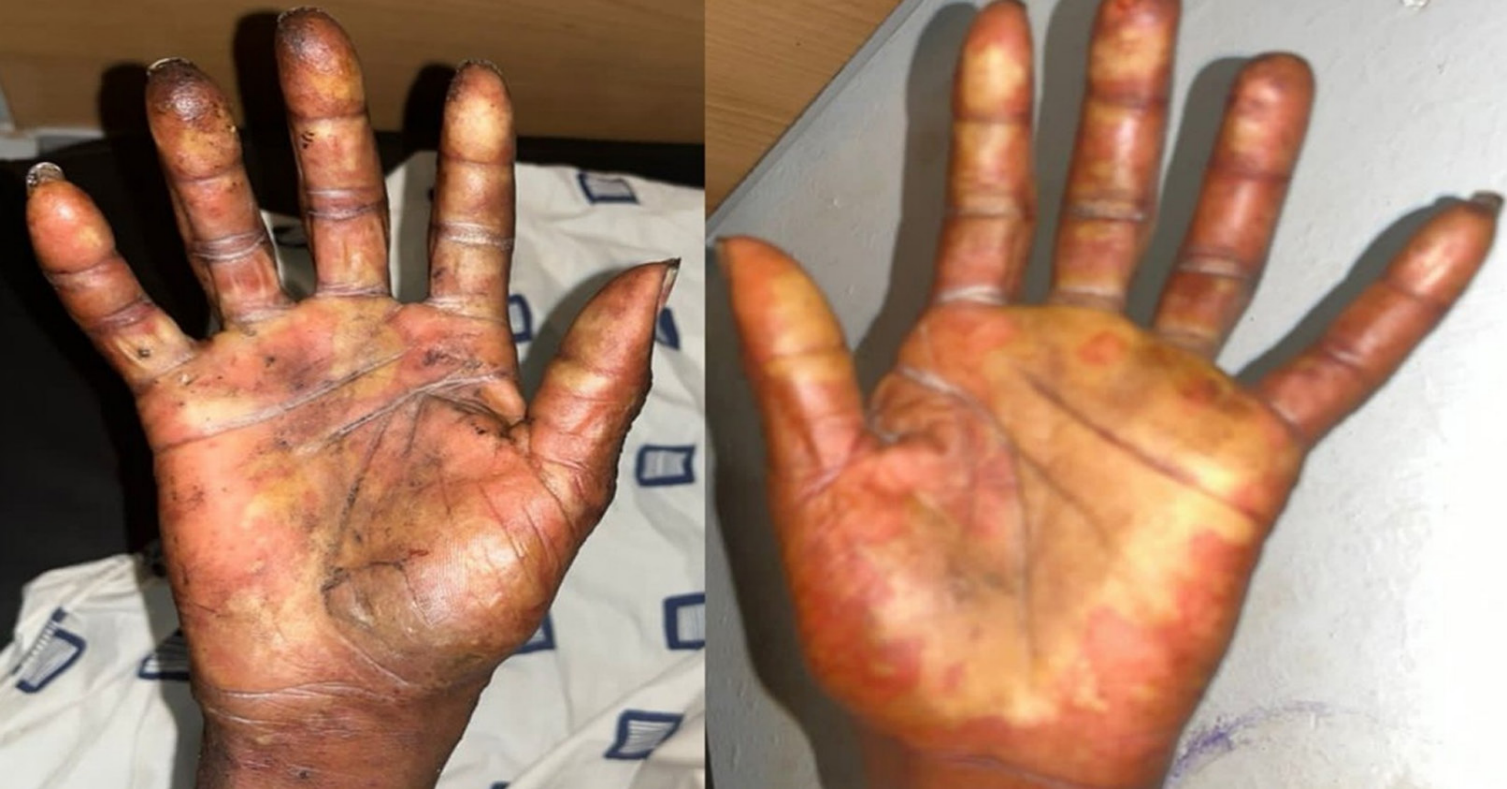
Mildly swollen palms with bullae and areas of erythema.

**FIGURE 3:**
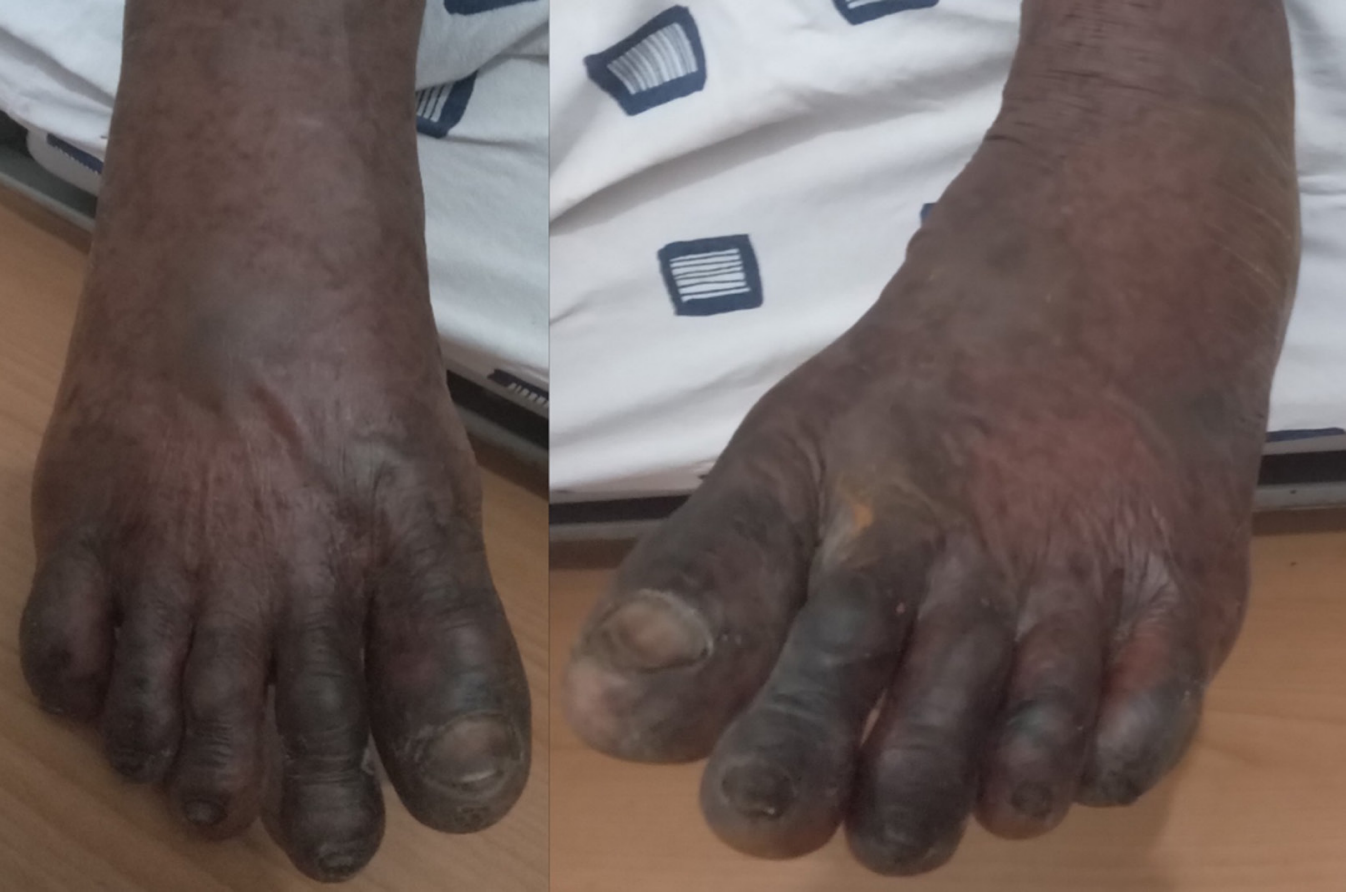
Mildly swollen feet with bullae and black necrotic skin overlying the dorsal aspects of the toes.

**FIGURE 4:**
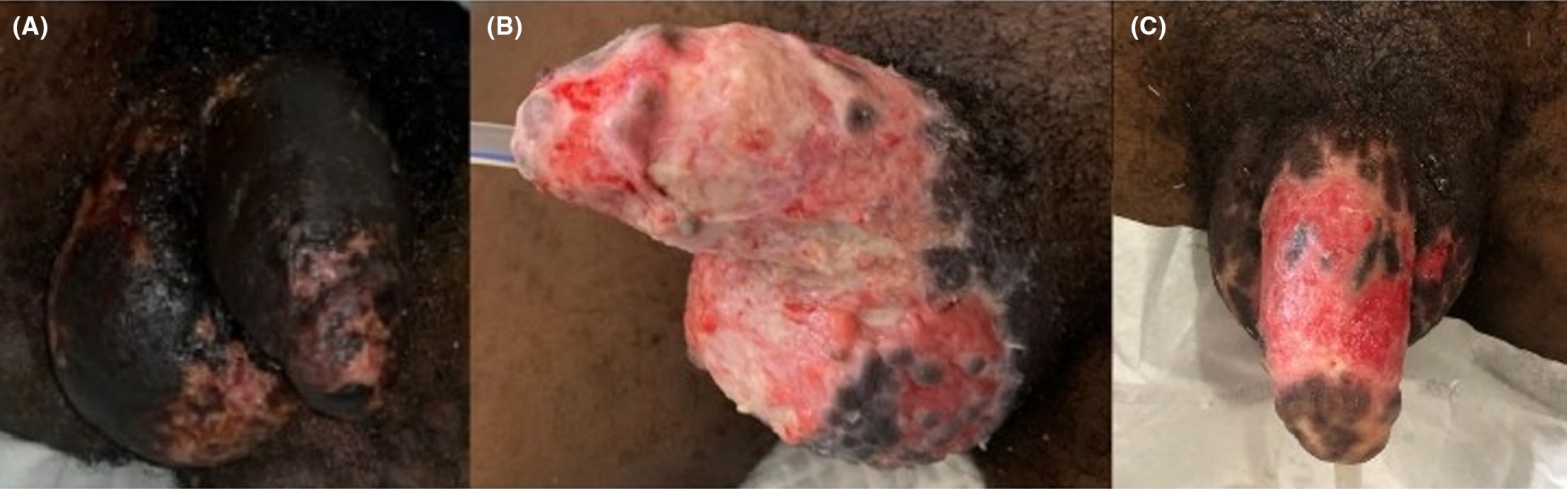
(A) Superficial ulceration of the scrotum and glans penis with overlying black necrotic skin (B) The appearance of the penis and scrotum after surgical debridement (C) Re‐epithelialization of the debrided scrotum and penis.

On physical examination, he was acutely ill‐looking, febrile (38.8°C), anicteric, not pale, not dyspneic, well‐nourished, well hydrated, and had no peripheral lymphadenopathy. He was alert, awake and oriented in time, place and person. His pulse and blood pressure were 136 beats per minute and 109/92 mmHg, respectively. He had a small linear abrasion on the lateral aspect of the left little finger (i.e., the bite site). There were multiple crusted vesicles measuring approximately 0.5 × 0.5 cm on the face (Figure 1A) without oral or nasal ulcers, conjunctival hyperemia or purulent discharge from the eyes. Bilaterally, he had vesicles which measured about 0.5 × 0.5 cm and bullae with sizes ranging from 1.5 × 2.0 cm to 2.5 × 4.5 cm on the extensor surfaces of the forearms, palms, knees, and dorsum of the feet. The palms and feet were mildly swollen, warm to touch and tender with areas of erythema (Figures 2 and 3). There was sparing of the anterior and posterior aspects of his trunk. Also noted was superficial ulceration of the scrotum and the glans penis with overlying black necrotic skin (Figure 4A). There was no crepitation on palpation of the affected areas. Sensation was intact in all the affected parts of the body.

### 2.2 Methods

Initial laboratory investigations showed neutrophilic leucocytosis, thrombocytopenia, minimally deranged renal biochemistries, elevated C‐reactive protein, normal liver biochemistries, and normal urinalysis as well as a negative HIV status. His random blood sugar was 8.7 mmol/L. Table 2 shows the results of the relevant laboratory tests that were done for the patient while on admission. The LRINEC score was 7 (Table 3) which was suggestive of a necrotizing infection. Accordingly, a diagnosis of squirrel bite with necrotizing soft tissue infection was made. He was started empirically on intravenous clindamycin 600 mg 8 hourly and ceftriaxone 2 g 12 hourly after taking a sample for blood culture. Intravenous fluids, acetaminophen, tramadol, anti‐tetanus serum and tetanus toxoid were administered. Additionally, rabies post‐exposure prophylaxis was provided by administering 1 mL of rabies vaccine intramuscularly on days 0, 3, 7, 14, and 28 together with infiltration of the bite site with human rabies immunoglobulin as he had not been previously vaccinated. Urologist was consulted for urgent debridement of the scrotum and penis (Figure 4B). A sample of necrotic tissue obtained intra‐operatively was sent for culture. Daily wound dressing with povidone‐iodine was started following debridement. Also, cleansing of the face and other areas with vesiculo‐bullous lesions using antiseptic solution was done on daily basis.

**TABLE 2:** Results of some laboratory tests for the patient.

Laboratory parameter	Initial result	Result on day 6 of admission	Result on day 11 of admission	Reference range
Total white cell count (×10^3^/uL)	11.59	22.93	8.14	4.0–10. 0
Neutrophil (×10^9^/uL)	9.48	18.93	6.21	1.5–7.0
Platelet (×10^9^/L)	70.0	336.0	322.0	150–450
Hemoglobin (g/dL)	12.6	6.3	10.2	11.0–16.0
Urea (mmol/L)	10.60	6.1		2.0–8.3
Creatinine (μmol/L)	135.0	97.0		60.0–120.0
Sodium (mmol/L)	133.7	135.8		135–145
Potassium (mmol/L)	3.52	4.1		3.5–5.5
Ionized calcium (mmol/L)	1.25	1.4		1.20–1.40
C‐reactive protein (mg/L)	200.0			0.0–10.0
Glycated hemoglobin (%)	4.3			4.0–5.9
Blood and necrotic tissue cultures	No bacterial growth			

**TABLE 3:** Computation of LRINEC score of the patient. Multiply glucose value in mmol/L by 18.015 to convert it to mg/dL. To convert creatinine from μmol/L to mg/dL, multiply its value by 0.0113.

Laboratory parameter	Patient's initial result	Patient's LRINEC point
Total white blood cell count (per μL)	11.59	0
Hemoglobin (g/dL)	12.6	1
Sodium (mmol/L)	133.7	2
Glucose (mg/dL)	156.7 (8.7 mmol/L)	0
Creatinine (mg/dL)	1.53 (135.0 μmol/L)	0
C‐reactive protein (mg/L)	200.0	4
Total score		7 (intermediate risk)

The patient complained of dizziness, palpitations, and had another fever spike (38.1°C) on the 6th day of admission. Repeat laboratory tests revealed a hemoglobin concentration of 6.3 g/dL. Results of blood and necrotic tissue cultures were negative despite further increases in the white blood cell and neutrophil counts as indicated in Table 2. He was transfused with two units of packed cells. Antibiotic regimen was empirically modified to a triple therapy consisting of intravenous ampicillin 2 g 6 hourly, gentamicin 80 mg 12 hourly and clindamycin 600 mg 8 hourly.

### 2.3 Conclusion and results

**FIGURE 5:**
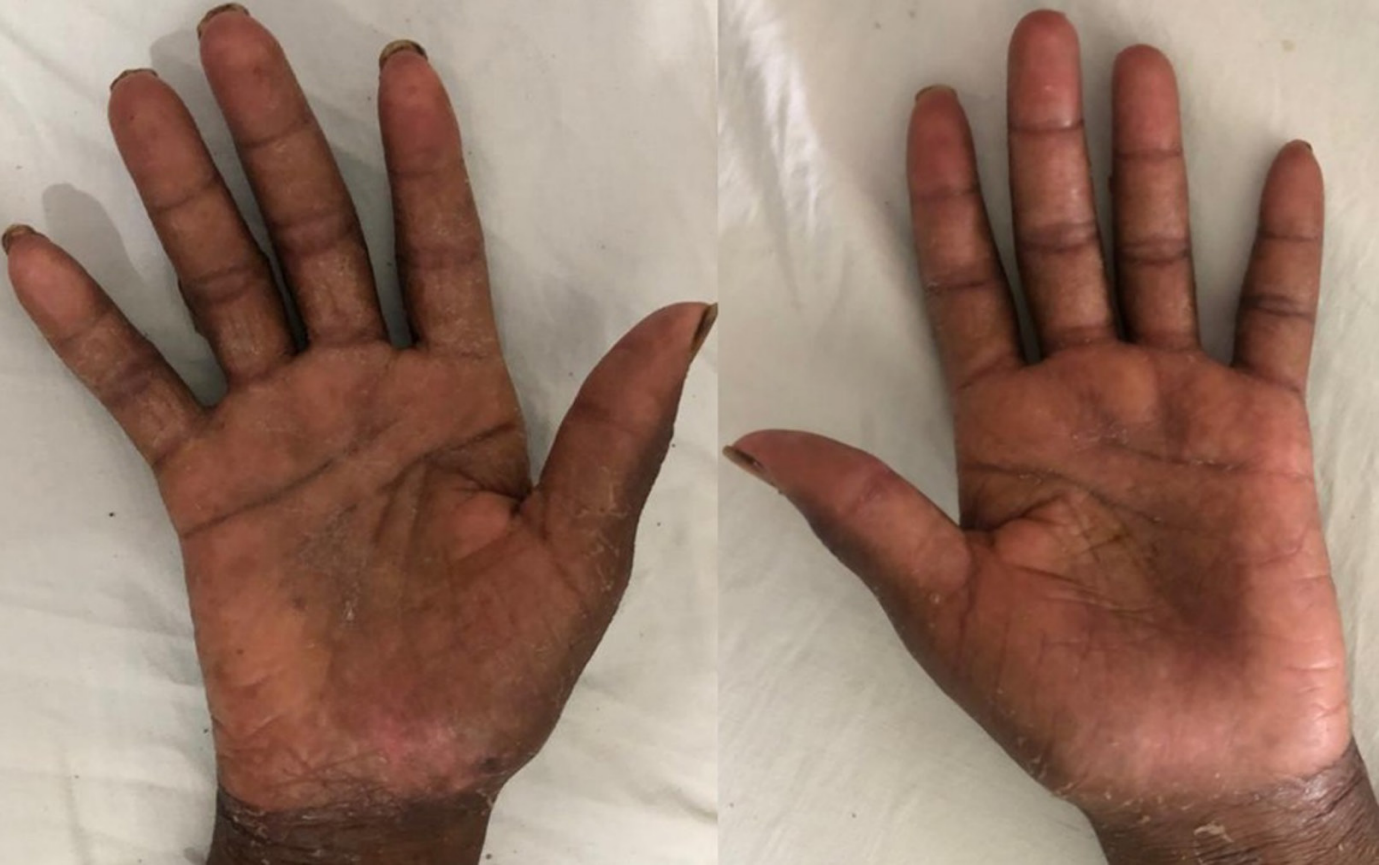
Resolution of bullae in the palms with dry skin completely peeled off.

**FIGURE 6:**
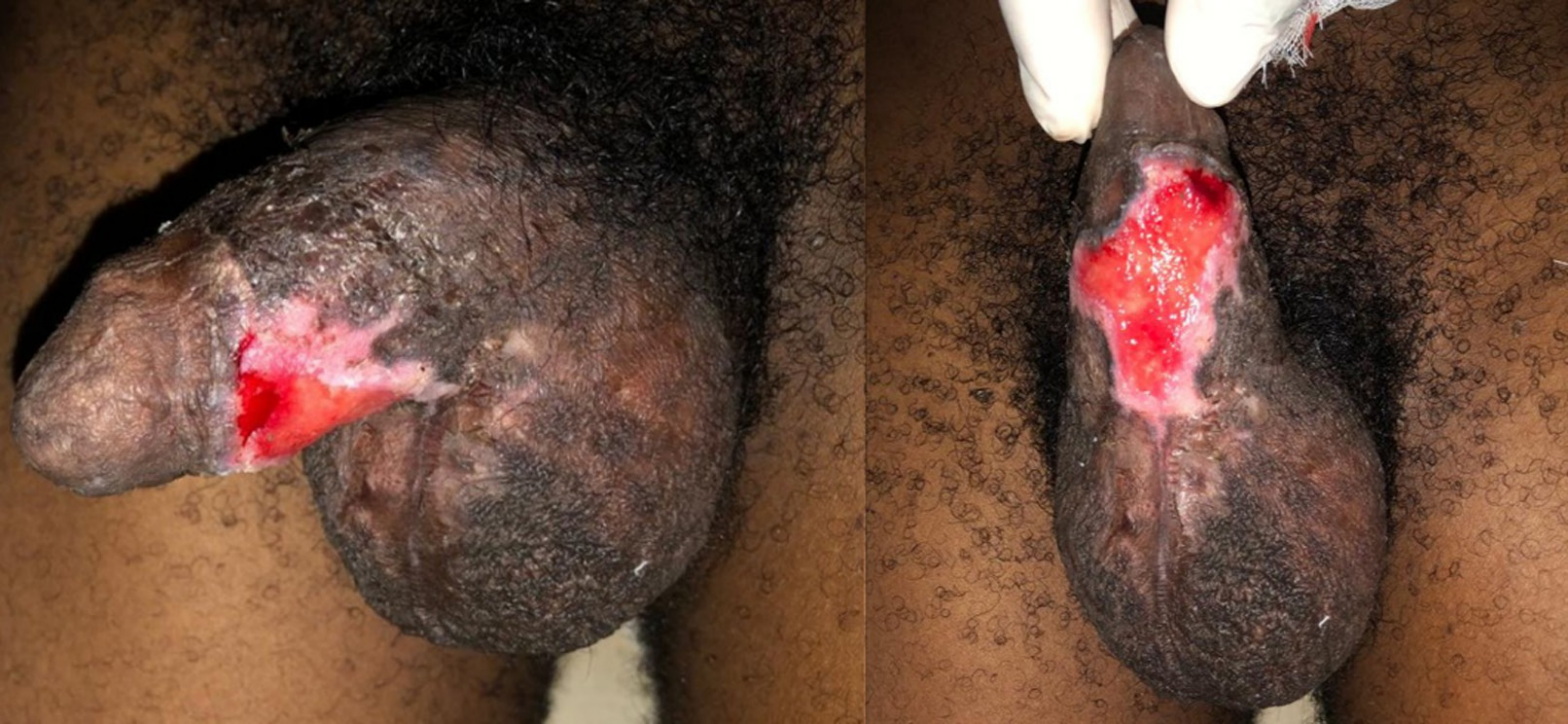
Nearly complete re‐epithelialization of the penis and scrotum 2 months after discharge.

The deranged renal biochemistries, thrombocytopenia, and hyponatremia which were evident from the initial laboratory evaluation had resolved by the 6th day of admission. A repeat complete blood count on the 11th day of admission showed normal white blood cell and neutrophil counts, with the hemoglobin level rising to 10.2 g/dL (Table 2). Over the ensuing days, the patient's condition improved satisfactorily. The crusted lesions on the face completely resolved (Figure 1B). Again, the vesicles and bullae on all the affected parts of his body healed with the dry skin peeling off (Figure 5). There was also gradual re‐epithelialization of the debrided scrotum and penis (Figure 4C). He was eventually discharged after 24 days of hospitalization. He was scheduled for regular follow‐up at the urology clinic and advised to continue wound dressing on an outpatient basis. At 2‐month follow‐up after discharge, there was nearly complete re‐epithelialization of the penis and scrotum (Figure 6).

## 3 DISCUSSION

In Ghana, squirrels and many other rodents are often hunted for their meat which is commonly served at traditional eateries that sell indigenous Ghanaian foods. Although some studies have suggested that squirrel bites are benign and seldom lead to local or systemic complications,^3^, ^12^ a few cases of these bites causing severe illness in humans and even death have been reported. To the best of our knowledge, this is the first reported case of necrotizing soft tissue infection involving the face, extremities, and external genitalia which occurred after a squirrel bite. The soft tissue destruction was mainly limited to the skin and subcutaneous tissue of the patient.

Even though the presence of multiple risk factors substantially predisposes one to developing necrotizing soft tissue infection,^8^ it can also occur in healthy individuals who do not have any risk factors^13^ as was noted in our patient. In India, a boy was reported to have had necrotizing fasciitis of the left lower limb after a squirrel bite over the dorsum of his left foot.^14^ Unlike our patient, there were no cutaneous eruptions or ulceration of the penis and scrotum. Aries et al described five patients from Ghana who presented with severe illness following squirrel bites. Three of them died immediately after the presentation. One was a 17‐year‐old boy who had a febrile illness which was associated with diarrhea, myalgia, and swollen hands as well as macules and vesicles on the face, shoulders and upper arms after his right thumb was bitten by a ground squirrel.^15^ These clinical manifestations are somewhat similar to those observed in our patient. In contrast to our case, the patient described by Aries et al also had hepatosplenomegaly and epistaxis but there was no perineal or lower extremity involvement. Again, two cases of a pyrexial illness associated with vesiculo‐papular skin eruptions occurring after the bite of the ground squirrel were reported in Nigeria.^16^ Other diseases which have been reported in association with squirrel bites include radial artery pseudoaneurysm,^17^ tularemia,^18^ anaphylactic shock,^19^ rabies,^20^ and lymphocutaneous sporotrichosis.^21^

Prompt initiation of broad‐spectrum empiric antibiotic therapy after taking a sample for culture, is one of the key principles in the management of necrotizing soft tissue infection. In monomicrobial infections, blood cultures are positive in about 60 percent of cases while in polymicrobial infections, the yield is approximately 20 percent.^4^ Although culture results were negative in the case of our patient, he responded well to our choice of triple antibiotic therapy. We administered rabies post‐exposure prophylaxis to the patient due to a remote risk of rabies transmission via squirrel bites.^20^

## 4 CLINICAL LEARNING POINT

Although squirrel bites are mostly benign and infrequently encountered in medical practice, they may lead to necrotizing soft tissue infections which can be life‐threatening. It is therefore advisable for individuals bitten by squirrels to seek medical attention for early detection and prompt treatment of any complication that may occur.

## AUTHOR CONTRIBUTIONS

**Prosper Adjei:** Conceptualization; formal analysis; investigation; methodology; writing – original draft; writing – review and editing. **Boateng Bosomtwi:** Writing – review and editing. **Prince Henry Asamoah:** Data curation. **Stanley Anenyemele Asasu:** Data curation. **Dominic Asante Ohene:** Data curation. **Augustina Amoakohene‐Yeboah:** Data curation. **Kojo Eduakwa Abban:** Data curation.

## FUNDING INFORMATION

The authors received no financial support for the authorship and/or publication of this article.

## CONFLICT OF INTEREST STATEMENT

The authors declare no potential conflicts of interest with respect to the authorship and/or publication of this article.

## ETHICS STATEMENT

Our institution does not require ethical approval for reporting individual cases or case series.

## CONSENT

Written informed consent was obtained from the patient to publish this report in accordance with the journal's patient consent policy.

## Data Availability

Data sharing is not applicable.

## References

[1] World Health Organization . Animal bites. 2024 Accessed March 13, 2024. https://www.who.int/news‐room/fact‐sheets/detail/animal‐bites

[2] Britannica . Squirrel. 2024 Accessed March 13, 2024. https://www.britannica.com/animal/squirrel

[3] Wyatt JP . Squirrel bites. BMJ. 1994;309:1694. 10.1136/bmj.309.6970.1694 PMC2542673 7819992

[4] Stevens DL , Baddour LM . Necrotizing soft tissue infections. Accessed March 13, 2024. https://www.uptodate.com/contents/necrotizing‐soft‐tissue‐infections 2024.

[5] Hakkarainen TW , Kopari NM , Pham TN , Evans HL . Necrotizing soft tissue infections: review and current concepts in treatment, systems of care, and outcomes. Curr Probl Surg. 2014;51:344‐362. 25069713 10.1067/j.cpsurg.2014.06.001 PMC4199388

[6] Stevens DL , Bryant AE . Necrotizing soft tissue infections. N Engl J Med. 2017;377:2253‐2265. 29211672 10.1056/NEJMra1600673

[7] Kalaivani V , Bharati VH , Indumathi VA . Necrotizing soft tissue infection‐risk factors for mortality. J Clin Diagn Res. 2013;7:1662‐1665. 24086868 10.7860/JCDR/2013/5535.3240 PMC3782925

[8] Headley AJ . Necrotizing soft tissue infections: a primary care review. Am Fam Physician. 2003;68:323‐328. 12892352

[9] Fujinaga J , Kuriyama A , Ikegami T , Onodera M . Laboratory risk indicator for necrotizing fasciitis score and patient outcomes. J Emerg Trauma Shock. 2021;14:38‐41. 33911435 10.4103/JETS.JETS_17_20 PMC8054806

[10] Wong CH , Khin LW , Heng KS , Tan KC , Low CO . The LRINEC (laboratory risk indicator for necrotizing fasciitis) score: a tool for distinguishing necrotizing fasciitis from other soft tissue infections. Crit Care Med. 2004;32:1535‐1541. 15241098 10.1097/01.ccm.0000129486.35458.7d

[11] Anaya DA , Dellinger EP . Necrotizing soft tissue infection: diagnosis and management. Clin Infect Dis. 2007;44:705‐710. 17278065 10.1086/511638

[12] Sriwiijittalai W , Wiwanitkit V . Squirrel bite: analysis of 35 cases. Ann Trop Med Public Health. 2017;10:478.

[13] Wong CH , Chang HC , Pasupathy S , Khin LW , Tan JL , Low CO . Necrotizing fasciitis: clinical presentation, microbiology, and determinants of mortality. J Bone Joint Surg Am. 2003;85:1454‐1460. 12925624

[14] Gupta S , Patil S , Gapchup R . Squirrel bite leading to necrotizing fasciitis. Pediatr Oncall J. 2020;17:19‐20.

[15] Aries MJ , Joosten H , Wegdam HH , van der Ven AJ . How innocent is the bite of a squirrel? Short report from central Ghana and literature review. Trop Dr. 2007;37:265‐266. 10.1258/004947507782332801 17988509

[16] McMillan B , Boulger LR . Squirrel bite fever. Trans R Soc Trop Med Hyg. 1968;62:567. 5691463 10.1016/0035-9203(68)90146-6

[17] Winter L , Chaudhry T , Wilson JL , Walker J , Huang D . Radial artery pseudoaneurysm from a squirrel bite. Cureus. 2023;15:e46080. 37900470 10.7759/cureus.46080 PMC10610304

[18] Borgschulte HS , Jacob D , Zeeh J , Scholz HC , Heuner K . Ulceroglandular form of tularemia after squirrel bite: a case report. J Med Case Rep. 2022;16:309. 35974355 10.1186/s13256-022-03510-8 PMC9381146

[19] Seshimo H , Ito T , Egusa C , et al. A case of anaphylactic shock induced by mealworm antigen in the bite of a Japanese flying squirrel. J Eur Acad Dermatol Venereol. 2021;35:e519‐e520. 33794057 10.1111/jdv.17265

[20] Kumari PL , Mohanan KR , Kailas L , Chacko KP . A case of rabies after squirrel bite. Indian J Pediatr. 2014;81:198. 23436194 10.1007/s12098-013-0990-2

[21] Saravanakumar PS , Eslami P , Zar FA . Lymphocutaneous sporotrichosis associated with a squirrel bite: case report and review. Clin Infect Dis. 1996;23:647‐648. 8879801 10.1093/clinids/23.3.647

